# Gender Differences in Young Adult Attitudes Towards Aging: A Qualitative Analysis of Aging Well Narratives

**DOI:** 10.1093/geroni/igaf122.3777

**Published:** 2025-12-31

**Authors:** Saahithi Punnam, Patricia Chilton, Matthias Mehl

**Affiliations:** University of Arizona, Tucson, Arizona, United States; St Mary’s College, St Mary’s College, Maryland, United States; University of Arizona, Tucson, Arizona, United States

## Abstract

This study explores young adults’ attitudes towards aging, with a focus on understanding their perceptions of older adulthood. While there is existing research that has examined perceptions of aging well among older populations, there is less data on how young adults conceptualize aging well and how these perceptions are formed. Our goal was to qualitatively identify themes in young adults’ aging well narratives and analyze gender differences in the prevalence of these themes. The sample consists of 99 students between ages 17 and 26 studying psychology at the University of Arizona. The participants were asked to provide a 4-minute free spoken response to the question, “In your own words, what does aging well mean to you?” in a Zoom interview. Themes and sub-themes were extracted using inductive analysis and represent a broad view of aging perceptions. The themes that emerged were physical health, physical appearance, elders as role models, mental health and fitness, social network, and attitudes of aging, life satisfaction, and well-being. MAXQDA software was used to identify gender differences in these themes. The largest gender differences emerged within the sub-themes of cognitive health, living life to the fullest, and elders as positive role-models. Female participants mentioned cognitive health and positive role models more than male participants, while male participants mentioned living life to the fullest more than females.

